# Disentangling nutritional pathways linking leafcutter ants and their co‐evolved fungal symbionts using stable isotopes

**DOI:** 10.1002/ecy.2431

**Published:** 2018-08-01

**Authors:** Jonathan Z. Shik, Winnie Rytter, Xavier Arnan, Anders Michelsen

**Affiliations:** ^1^ Centre for Social Evolution Department of Biology University of Copenhagen Universitetsparken 15 2100 Copenhagen Denmark; ^2^ Smithsonian Tropical Research Institute Apartado 0843‐03092 Balboa Ancon Republic of Panama; ^3^ CREAF Cerdanyola del Vallès ES‐08193 Catalunya Spain; ^4^ Terrestrial Ecology Section Department of Biology University of Copenhagen Universitetsparken 15 2100 Copenhagen Denmark

**Keywords:** ^13^C, ^15^N, attine ants, carbon and nitrogen isotopes, nutritional ecology, tropical rainforest

## Abstract

Leafcutter ants are the ultimate insect superorganisms, with up to millions of physiologically specialized workers cooperating to cut and transport vegetation and then convert it into compost used to cultivate co‐evolved fungi, domesticated over millions of years. We tested hypotheses about the nutrient‐processing dynamics governing this functional integration, tracing ^15^N‐ and ^13^C‐enriched substrates through colonies of the leafcutter ant *Atta colombica*. Our results highlight striking performance efficiencies, including rapid conversion (within 2 d) of harvested nutrients into edible fungal tissue (swollen hyphal tips called gongylidia) in the center of fungus gardens, while also highlighting that much of each colony's foraging effort resulted in substrate placed directly in the trash. We also find nutrient‐specific processing dynamics both within and across layers of the fungus garden, and in ant consumers. Larvae exhibited higher overall levels of ^15^N and ^13^C enrichment than adult workers, supporting that the majority of fungal productivity is allocated to colony growth. Foragers assimilated ^13^C‐labeled glucose during its ingestion, but required several days to metabolically process ingested ^15^N‐labeled ammonium nitrate. This processing timeline helps resolve a 40‐yr old hypothesis, that foragers (but apparently not gardeners or larvae) bypass their fungal crops to directly assimilate some of the nutrients they ingest outside the nest. Tracing these nutritional pathways with stable isotopes helps visualize how physiological integration within symbiotic networks gives rise to the ecologically dominant herbivory of leafcutter ants in habitats ranging from Argentina to the southern United States.

## Introduction

Social insects channel vast amounts of resources through their colonies at a global scale (Brian 1978, Del Toro et al. 2012, Griffiths et al. 2018). However, while ant foraging is a conspicuous sight in most terrestrial habitats (Lanan 2014), the fates of resources inside ant nests are rarely observed (Tschinkel 1991, 2011). Moreover, while the basic details of colony growth are well known, from queen‐laid eggs, across several larval instars, pupation, and the adult worker life cycle (Oster and Wilson 1978), the underlying nutrient processing dynamics are described for few of the *>*14,000 extant ant species. Dietary tracer experiments using foods labeled with heavy isotopes of carbon, phosphorus and nitrogen have enabled researchers to trace the flow of labeled resources as they flow among colony members inside nests where allocation dynamics are difficult to directly observe (e.g., Howard and Tschinkel 1981, Feldhaar et al. 2010, Hölldobler and Kwapich 2017).

Radioactive tracers were the primary tool in isotopic research about resource allocation within colonies for over 60 yr (Wilson and Eisner 1957, Golley and Gentry 1964, Markin 1970, Sorensen and Vinson 1981), but stable isotope natural abundance studies of nitrogen (^15^N) and carbon (^13^C) are now commonly used to infer dietary habits when foraging dynamics occur out of sight (Davidson et al. 2003), when species are either rare (Jacquemin et al. 2014) or are members of diverse communities (e.g., Blüthgen et al. 2003, Smith and Suarez 2010, Penick et al. 2015), and when colonies are distributed across large spatial (Tillberg et al. 2007, Wilder et al. 2011) and temporal scales (Mooney and Tillberg 2005, Yang 2006, Roeder and Kaspari 2017). Stable isotope enrichment experiments also provide powerful tools for visualizing nutrient exchange among symbiotic partners (Kiers et al. 2011), making such experiments useful in ant ecology since ants often rely on nutrients derived from hemipterans (Shik et al. 2014a), plants (Sagers et al. 2000, Fischer et al. 2005, Pinkalski et al. 2018), and microbes (Feldhaar et al. 2007, Pinto‐Tomás et al. 2009, Sapountzis et al. 2015).

### Ecology of farming productivity

Leafcutter ants of the genus *Atta* are ideally suited for isotopic experiments because they farm a co‐evolved fungal symbiont for food, harvesting fresh vegetation and using it to produce fungal crops in massive underground nests that can feed millions of workers (Hölldobler and Wilson 2010). Fungal symbionts are fully integrated parts of the leafcutter ant digestive system that begin to process harvested resources when gardener ants deposit mixtures of chewed vegetation and digestive enzymes on top of the fungus garden (Moller et al. 2011). Fungal symbionts (De Fine Licht et al. 2013), gardener ants (Quinlan and Cherrett 1979), and developing ant larvae (Erthal et al. 2007) then collectively convert this composted substrate into structural fungal hypha and edible gongylidia, swollen hyphal tips that concentrate nutrients and grow in bundles called staphyla (Martin et al. 1969, Quinlan and Cherrett 1979, Mueller et al. 2001, Schiøtt et al. 2010). We measured these production dynamics with novel sampling resolution, allowing foragers in laboratory colonies of *A. colombica* to harvest isotopically‐enriched substrate, and then traced two isotopically labeled compounds (^13^C‐enriched glucose and ^15^N‐enriched ammonium nitrate) through symbiotic networks across over 800 samples spanning 20 d. Below, we outline how this methodology enabled us to test hypotheses about nutrient integration through the fungus garden (across layers of hyphae, within edible tissues, and disposal in the trash), allocation among ant consumers (adult and immature castes), and processing within individual ants (transported or assimilated).

Based on the timing of isotopic enrichment within hyphae at vertical layers of fungus, we first tested a *fungus layers* hypothesis, previously inferred from patterns of enzyme activity in leafcutter fungus gardens of serial downward nutrient integration within the garden (Moller et al. 2011, De Fine Licht et al. 2013). A vertical processing dynamic implies an organizing principle whereby workers systematically deposit fresh vegetation at the top of the fungus garden to initiate its use in the cultivation process. We next compared enrichment across fungal tissues to test a *fungus food* hypothesis: nutrient integration is targeted towards food production (edible gongylidia) rather than biomass of the non‐differentiated hypha surrounding the gonyglidia. We further explored waste disposal dynamics, sampling trash piles to quantify overall processing rates of nutrients following their integration into the fungus garden. Since fungal cultivars grow best on specific nutritional blends (Shik et al. 2016), we tested a *waste disposal* hypothesis, that a potential mechanism of meeting their cultivar's nutritional needs is that ant farmers select specific nutrients from the composted substrate initially provided to their cultivars through nutrient‐specific disposal of harvested substrates.

### Nutrient allocation

Transitioning from the fungal cultivar to the ant consumers, we next traced labeled compounds as they were ingested and allocated among physiologically specialized ant castes. Foraging ants are generally assumed to be maintained primarily by carbohydrates (Markin 1970, Sorensen and Vinson 1981), but they must forage to also satisfy nutritional requirements of non‐foraging nestmates, including larvae whose growth depends on protein acquisition (Dussutour and Simpson 2008a). Still, most nutrient allocation decisions may actually occur inside the nest, as ants regurgitate ingested liquids from specialized abdominal storage organs (*hereafter* ‘gasters’) and share them with nestmates (Cook and Davidson 2006). Thus, ingestion does not guarantee assimilation in ants, and we hypothesized that carbohydrates would be preferentially retained by adult workers and proteins would be shunted through the fungus garden and towards developing larvae.

We tested this allocation hypothesis by comparing isotope enrichment of two types of nutrients among ant castes: a carbohydrate (^13^C‐enriched glucose), and a source of the nitrogen used to build proteins (^15^N‐enriched ammonium nitrate). We tested the prediction among ant consumers that adult workers (foragers and gardeners) have higher mean ^13^C values and developing brood (larvae and pupae) have higher mean ^15^N values. We then compared enrichment timelines across castes to test whether garden‐inhabiting castes (gardeners and larvae) assimilate nutrients received directly from returning foragers or only later, ostensibly after they had been processed through the fungus garden.

### Nutrient processing

Like microbial symbionts of other insects (e.g., bees, Engel et al. 2012, termites, Poulsen et al. 2014), fungal cultivars and their associated bacteria convert difficult to digest compounds (e.g., plant cellulose, Moreira‐Soto et al. 2017) and inaccessible molecules (e.g., atmospheric N_2,_ Pinto‐Tomás et al. 2009) into metabolically useful nutrients for their leafcutter ant hosts. Despite these derived symbiont processing services, adult leafcutter ants are thought to ingest plant sap while cutting leaves outside the nest (Littledyke and Cherrett 1976), and bypass fungi for 90% of their energy and nutrient requirements (Quinlan and Cherrett 1979, Bass and Cherrett 1995).

We propose the function of such plant sap foraging remains unclear, since leafcutters produce their fungal crops by vectoring ingested liquids from their gasters to fungal cultivars in fecal droplets (Martin and Martin 1970, Schiøtt et al. 2010, De Fine Licht et al. 2013). We tested two resource processing hypotheses about whether and when foraging leafcutter ants assimilate ‘wild caught’ resources, separating ant gasters prior to isotope analyses to distinguish between two types of processing dynamics: *fungus‐first* (ingested nutrients transported in the gaster) and *forager‐first* (ingested nutrients directly assimilated in head‐thorax tissue) (*as per* Tillberg et al. 2006, Feldhaar et al. 2010). We compared the timing of enrichment across ant tissues, assuming that simultaneous ingestion (gaster enrichment) and assimilation (head‐thorax enrichment) indicates forager‐first nutrient processing without intermediate processing by fungal cultivars. We then tested whether these processing dynamics depend on compound digestibility, with forager‐first processing of glucose (e.g., it can be directly used to fuel metabolic respiration or converted to glycogen and stored in fat body cells, Arrese and Soulages 2010), and fungus‐first processing of the less readily metabolized compound ammonium nitrate. Finally, we compared nutrient processing between foragers and gardeners, a non‐foraging caste we predicted would have greater reliance on fungus‐first resource acquisition.

## Methods

### Colonies of Atta colombica

We established queenless subcolonies (*hereafter* colonies) from five large queenright colonies of the leafcutting ant *Atta colombica* collected in Panama from 2009 to 2012 and maintained at the University of Copenhagen in a climate‐controlled room (25°C, 70% RH, minimal daylight). For four months prior to the experiment, colonies were fed leaves, apples, and rice three times per week (provided in small removable trays) and were housed under inverted beakers in open plastic nest boxes (38 × 28 cm) with fluon‐coated walls that remained connected via tygon tubing to the queenright nest chamber in the central nest box (Appendix S1). This was done in order to avoid isotopic contamination in the central nest box, and mimicked natural colonies where colonies typically have many nest chambers connected directly or indirectly to a central chamber containing the queen and can regulate the flow of resources and nestmates among chambers. Experimental colonies were separated from the central nestbox just prior to the start of the experiment. Examination of trash piles and fungus gardens during the experiment, and demographic analyses performed after the experiment indicated colonies experienced low worker mortality (i.e., few dead workers were found) and high fungus garden stability (i.e., colonies continuously produced new fungus) during the 20‐d isotopic sampling period (albeit with diminished larvae numbers by the end because colonies lacked queens), with (mean ± SE) 20.7 ± 5.3 g fungus (dry mass), 11,520 ± 2,401 workers, 131 ± 55 larvae, and 1,599 ± 423 pupae per colony (Appendix S2: Table S1).

### Isotopically enriched diets

We provided five colonies with isotopically enriched diet and traced the single pulse of ^13^C and ^15^N enrichment from this foraging event through colonies over 20 d. We modified 1:3 and 3:1 protein:carbohydrate (P:C) agar‐based diets from Dussutour and Simpson (2008b) (with a 60 g/L protein plus carbohydrate dilution), to be enriched with ^13^C (D‐glucose: ^13^C_6_H_12_O_6_, Sigma‐Aldrich) and ^15^N (ammonium nitrate: ^15^NH_4_^15^NO_3_, Sigma‐Aldrich). For detailed recipe information, see Appendices S1 and S2: Table S2. We used these diets as the means of isotopic enrichment because ants harvest a variety of plant‐based resources in nature, including plant nectar (Littledyke and Cherrett 1976) and fallen fruit (Evison and Ratnieks 2007), and because the diets gave us precise and replicable control over the amount of isotopic enrichment. Moreover, these diets enabled ants to successfully integrate the nutrients into their farming systems, with ants licking the diets and also cutting pieces and planting them on their gardens (Shik et al. 2016). Isotopic analyses of ^13^C and ^15^N (Atom Percent Excess, APE) values, *see below*) indicated enrichment for the 1:3 P:C diet of 1.9% ^13^C and 6.9% ^15^N, and enrichment values for the 3:1 P:C diet of 2.4% ^13^C and 4.2% ^15^N (see Appendices S1 and S2: Table S2 for details). These enrichment values were found, in pilot trials, to optimize isotope detection in colony components.

On Day 1 of the experiment, colonies were allowed to forage between diets with 1:3 and 3:1 P:C ratios for 24 h and select their own P:C intake target (Behmer 2009). Workers harvested substantial amounts of enriched diets (± SE): 46.5 (± 17.9)% of the initial weight and 0.64 (± 0.26) g dry mass of 1:3 P:C diet and 19.2 (± 11.0)% of the initial weight and 0.29 (± 0.16) g dry mass of 3:1 P:C diet (Appendix S2: Table S1). This *initial diet harvest* was measured for each colony and used as a covariate in subsequent statistical analyses of isotope enrichment. Following the Day 1 pulse, colonies were fed unenriched 1:3 and 3:1 P:C diets (Days 2–6) and bramble leaves (Days 7–20) whose ^13^C and ^15^N levels were at the natural abundance level (Appendix S1).

### Sample collection

**Figure 1:**
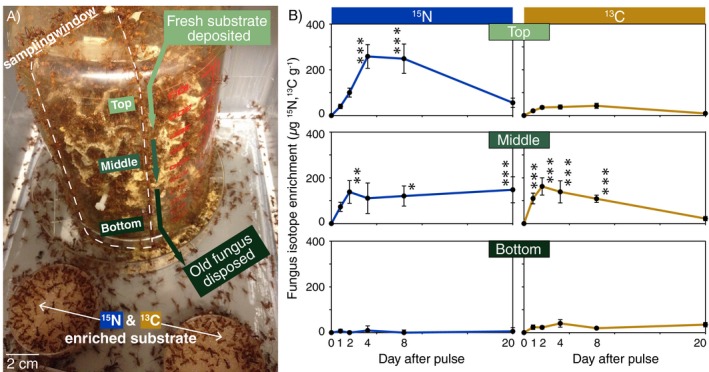
(A) The experimental setup used to determine that (B) Enrichment timelines generally support downward nutrient integration across layers of fungal hyphae, while also highlighting key nutrient‐specific processing dynamics. On Day 1, colonies were provided with nutritionally defined substrate enriched with known amounts of ^15^N (blue) and ^13^C (gold). Colonies cultivated their fungus inside inverted beakers, and samples were collected through a removable sampling window (dashed white outline) on days 0, 1, 2, 4, 8, and 20 after the isotopic initial pulse. Enrichment units (μg ^15^N and ^13^C/g ± SE) are relative to the natural abundance (measured on Day 0) per gram dry mass of sampled tissue. Tukey test results are only shown in panels with significant time effects, where significant differences (*P < 0.05, **P < 0.01, ***P < 0.001) indicate days differing significantly from day 0 (no significant differences existed within enrichment values differing from day 0).

We sampled 5 colonies on the day before the isotopic pulse (Day 0, natural abundance), and again on days 1, 2, 4, 8, and 20 following the pulse (Appendix S1). Nests were fit with removable ‘collection windows’ enabling non‐disruptive sampling within the fungus garden (Fig. 1A). On each sampling day, we collected fungal hyphae (with gongylidia removed) from top, middle, and bottom layers of the garden (*as per* Moller et al. 2011), and collected gongylidia (packed in tiny 0.5 mm diameter) bundles called staphyla from the middle garden layer where they were most abundant (De Fine Licht et al. 2014). We removed trash piles at each feeding event and analyzed homogenized trash pile samples, when available, on each sampling day. We collected adult ants in two groups: foragers (large and medium‐sized workers collected outside the nest) and gardeners (small ants collected inside fungus chambers) (Wilson 1980, Forti et al. 2012). Prior to isotopic analyses, these ants were anesthetized at 4°C and divided into gaster and head‐thorax samples. We also collected larvae and pupae in the middle layer of fungus gardens where they were most abundant, analyzing whole bodies in single samples as they could not be readily separated into gaster and head‐thorax samples as in the adults. Overall, from each colony at each sampling event, we collected the following samples for isotopic analyses: foragers (*n* = 4) and gardeners (*n* = 2), larvae (*n* = 3), pupae (*n* = 3), fungal hypha at three layers (*n* = 3 per layer), fungal gongylidia (*n* = 1 in the middle layer), and trash pile (*n* = 2), to yield 900 planned isotopic samples (Appendices S1 and S2: Table S3), and 840 actual isotopic samples (Appendix S2: Table S4A, B).

### Stable isotope analyses

Samples were dried at 60°C for ≥24 h, homogenized, weighed into tin capsules, and analyzed for ^15^N/^14^N and ^13^C/^12^C using a Eurovector CN analyzer (Pavia, Italy) coupled to an Isoprime (Cheadle Hulme, UK) mass spectrometer. Natural abundances of ^15^N and ^13^C provided a baseline for interpreting subsequent enrichment, and were determined from Day 0 samples using the equations provided below (Fischer et al. 2005, Fry 2006), where peach leaves (NIST RM 1547) were used as the internal spectrometry calibration standard for N and C (*as per* Brand et al. 2014), and where reference gas was calibrated against international standards IAEA C5, CH6, CH7, N1, N2 and USGS 25, 26, 32:

$$\begin{array}{cc} \delta \;{}^{15}N\;\left[‰\text{vs}.\text{at‐air}\right]\;=\;\; & [\left({}^{15}N/{}^{14}N_{\mathrm{sample}}\right)/\left({}^{15}N/{}^{14}N_{\mathrm{standard}}\right)-1]\;\\ & \times \;1,000 \end{array}$$

$$\begin{array}{cc} \delta {}^{13}C\;\left[‰\;\text{vs}.\;\text{V‐PDB}\right]\;= & \;\;[\left({}^{13}C/{}^{12}C_{\mathrm{sample}}\right)/\left({}^{13}C/{}^{12}C_{\mathrm{standard}}\right)-1]\;\\ & \times \;1,000 \end{array}$$

We next calculated Atom Percent (at%) of ^15^N and ^13^C as the percentage of heavy isotope moles of N or C in a sample, and Atom Percent Excess (APE) as the at% ^15^N or ^13^C of enriched samples above the Day 0 natural abundance:

$$\text{APE}{}^{15}N\;=\;\text{at}\%{}^{15}N_{\mathrm{sample}}-\text{at}\%{}^{15}N_{\mathrm{Natural}\mathrm{abundance}}$$

$$\text{APE}{}^{13}C\;=\;\text{at}\%{}^{13}C_{\mathrm{sample}}-\text{at}\%{}^{13}C_{\mathrm{Natural}\mathrm{abundance}}$$

For statistical analyses, we calculated excess μg ^15^N and μg ^13^C per gram dry mass of each sample (*hereafter* ^15^N and ^13^C) from the APE and the sample dry mass:

$$\mu g{}^{15}\text{N per}\;\text{g dry}\;{\text{mass}}^{-1}\;=\;\text{APE}\;\times \;\text{sample}\;{\text{mass}}^{-1}\;\times \;1,000$$

$$\mu g{}^{13}\text{C per}\;\text{g dry}\;{\text{mass}}^{-1}\;=\;\text{APE}\;\times \;\text{sample}\;{\text{mass}}^{-1}\;\times \;1,000$$

### Statistical analyses

#### Ecology of farming productivity

*Fungus layers:* We performed a mixed model analysis using the lme function in the nlme package (Pinheiro et al. 2018) in R 3.2.4 (R Development Core Team 2016) to compare enrichment timelines of ^15^N and ^13^C (*i.e.,* APE values) across layers of fungal hyphae where time (Day 0, 1, 2, 4, 8, 20; a categorical variable), nutrient (^15^N and ^13^C), layer (top, middle, bottom) and their interactions were fixed factors. Initial diet harvest was included as a covariate, as colonies varied in their Day 1 harvest of enriched diet (0.05 to 2.1 g dry mass; Appendix S2: Table S1). We controlled for temporal repeated measures (*i.e.,* the same layer from a given colony was sampled across days) and spatially repeated measures (*i.e.,* the same sample from a given layer and colony was analyzed for both ^15^N and ^13^C) by including as random factors sample ID nested within layer, which in turn was nested within colony ID. *Fungus food:* We compared nutrient integration in middle‐layer gongylidia with surrounding middle‐layer hyphae by performing a mixed model analysis where time, nutrient, tissue (hyphae, gongylidia) and their interactions were fixed factors, initial diet harvest was a covariate, and colony ID was a random factor. Since one gongylidia sample and three hyphal samples were collected per layer, colony and day, we analyzed mean hyphal enrichment values to generate a balanced model. *Waste disposal:* We used a mixed model to analyze trash‐pile samples for ^15^N and ^13^C enrichment, with time, nutrient, and their interaction as fixed factors, initial diet harvest as a covariate, and sample ID nested in colony ID as a random factor.

To facilitate direct statistical comparisons of ^15^N and ^13^C enrichment, we standardized the data prior to each analysis by calculating *Z* scores separately for ^13^C and ^15^N data. When significant differences existed among ^15^N and ^13^C enrichment (*i.e.,* significant ‘nutrient’ main or interaction effects)*,* we plotted observed nutrient means (± SE), as they generated similar temporal patterns as *Z*‐scores, and enabled comparison with other similar published results. Otherwise, we plotted the *Z*‐scores combining the means of ^15^N and ^13^C data. In all cases, we interpreted significant differences using posthoc Tukey tests.

#### Nutrient allocation

We compared ^15^N and ^13^C enrichment across adult ants and brood, performing mixed model analyses in SAS (V9.4, Proc GLIMMIX) with time (Day 1, 2, 4, 8, 20), nutrient, caste (forager, gardener, larva, pupae), and their interactions as fixed factors, initial diet harvest as a covariate, and caste nested in colony ID as a random factor. We analyzed adult ant head‐thorax tissue as the allocation hypothesis focused on assimilated nutrients, and analyzed *Z*‐scores, using posthoc Tukey tests to interpret significant differences among castes within sampling days.

#### Nutrient processing

We used a mixed model (proc GLIMMIX) testing for differences among tissues within ants over time. We performed separate analyses for ^15^N and ^13^C enrichment, with time, caste (forager, gardener), and tissue (gaster, head‐thorax) as fixed factors, and the random factors colony ID, time × colony ID, and individual ID nested in (time × colony ID). This analysis also modeled within‐subject tissue effects (gaster vs. head‐thorax) as a repeated measure for organs within individuals. Separate analyses for ^15^N and ^13^C enrichment were preferred for nutrient processing analyses, given the overall complexity of the model, and our focus on interpreting nutrient processing timelines within ants. To test for latency between ingestion (enrichment of gaster) and assimilation (enrichment of head‐thorax), we used post‐hoc Tukey tests to interpret significant differences within tissues across days and across tissues within sampling days.

## Results

### Ecology of farming productivity

Within minutes of placing diets inside nest boxes, foragers could be observed licking and cutting agar‐based substrates, and then carrying them back to their nests (Fig. 1A). This initial diet harvesting effort significantly influenced subsequent fungus enrichment levels (Table 1). *Fungus layers:* Nutrients exhibited distinct downward enrichment timelines within and across vertical layers of the fungus garden (Time × nutrient × layer interaction effects in Table 1, Fig. 1B). First, ^15^N trended upwards in both the top and middle layers on the day of harvest, becoming significantly enriched in the middle layer by day 2 and in the top layer by day 4. In contrast, ^13^C was directly integrated in the middle layer, where it became significantly enriched by the first day (Fig. 1B). Second, ^15^N levels remained steady in the middle layer over 20 d (Fig. 1B), while ^13^C became significantly depleted in the middle layer by Day 20 (Fig. 1B). Despite these middle layer depletion differences, neither isotope was detected at significant levels in the bottom layer over 20 d (Fig. 1B).

**Table 1:** Statistical tests about the ecology of farming productivity, and about how leafcutter ant consumers allocate and process nutrients, based on an isotope enrichment feeding experiment

Test		Source	Num df	Denom df	*F* value	*P* value
Fungus layers		Intercept	1	236	0.00	0.985
		Time	5	224	16.81	0.0001
		Nutrient	1	236	0.00	1.000
		Layer	2	8	6.90	0.018
		Time × nutrient	5	236	11.33	0.0001
		Time × layer	10	224	5.05	0.0001
		Nutrient × layer	2	236	60.46	0.0001
		Time × nutrient × layer	10	236	16.09	0.0001
		Initial diet harvest	1	3	19.85	0.021
Fungus food		Intercept	1	91	0.00	0.992
		Time	5	91	7.81	0.0001
		Nutrient	1	91	0.00	0.999
		Tissue	1	91	18.27	0.0001
		Time × nutrient	5	91	0.39	0.855
		Time × tissue	5	91	2.40	0.043
		Nutrient × tissue	1	91	1.99	0.162
		Time × nutrient × tissue	5	91	0.39	0.855
		Initial diet harvest	1	3	47.02	0.006
Waste disposal		Intercept	1	73	0.00	0.965
		Time	5	73	7.42	0.0001
		Nutrient	1	73	0.00	1.000
		Time × nutrient	5	73	0.03	0.999
		Initial diet harvest	1	3	0.45	0.550
Nutrient allocation		Time	4	64.4	9.93	0.0001
		Nutrient	1	452	0.07	0.799
		Caste	3	15	7.10	0.003
		Time × nutrient	14	452	3.81	0.005
		Time × caste	12	64.3	5.17	0.0001
		Nutrient × caste	3	452	4.38	0.005
		Time × nutrient × caste	12	452	1.31	0.207
		Initial diet harvest	1	15	36.77	0.0001
Nutrient processing	^15^N	Time	5	35.86	2.40	0.056
		Caste	1	129.9	11.14	0.001
		Tissue	1	147.2	30.83	0.0001
		Time × caste	5	129.8	1.50	0.193
		Time × tissue	5	147.2	2.69	0.024
		Caste × tissue	1	147.2	6.65	0.011
		Time × caste × tissue	5	147.2	1.49	0.196
	^13^C	Time	5	64	6.23	0.0001
		Caste	1	139.9	15.76	0.0001
		Tissue	1	176.1	35.90	0.0001
		Time × caste	5	140	2.41	0.039
		Time × tissue	5	176.1	3.32	0.007
		Caste × tissue	1	176.1	0.32	0.575
		Time × caste × tissue	5	176.1	0.58	0.719

*Notes*

*Fungus layers:* We compared enrichment timelines of ^15^N and ^13^C across vertical layers of fungal hypha, using a mixed model where time (categorical variable: Day 0, 1, 2, 4, 8, 20), nutrient (^15^N and ^13^C), and layer (top, middle, bottom) were fixed factors, initial diet harvest was a covariate, and sample ID nested within layer and then nested within colony ID were random factors. *Fungus food:* We compared enrichment in gongylidia relative to surrounding middle layer hypha, using a mixed model where time, nutrient, and tissue (hyphae, gongylidia), were fixed factors, initial diet harvest was a covariate and colony ID was a random factor. *Waste disposal:* We used a mixed model analysis comparing ^15^N and ^13^C enrichment in trash piles, with time, nutrition and their interaction as fixed factors, initial diet harves as a covariate, and sample nested in colony ID as a random factor. *Nutrient allocation:* We used a mixed model analysis comparing isotope enrichment across castes, with time (excluding Day 0), nutrient, caste (forager, gardener, larva, pupae) and their interactions as fixed factors, initial diet harvest as a covariate, and caste nested in colony ID as a random factor. *Nutrient processing:* We examined ^15^N and ^13^C enrichment within ants, using separate models for ^15^N and ^13^C with time, caste (forager, gardener), tissue (head‐thorax, gaster), and their interactions as fixed factors, and the random factors colony ID, Day × colony ID, and individual ID nested in (Day × colony ID). This analysis also included within‐subject tissue effects (gaster vs. head‐thorax), using a repeated statement for organs within individuals.

**Figure 2:**
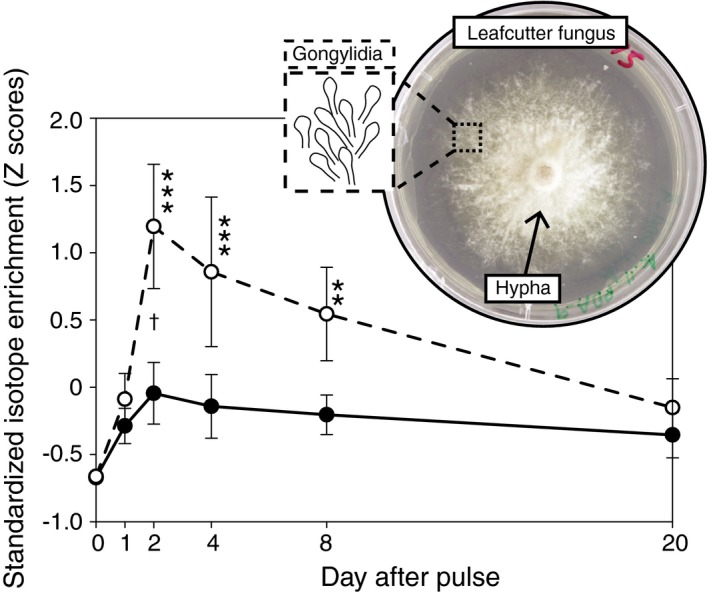
Enrichment timelines indicate rapid and targeted enrichment of edible gongylidia relative to surrounding structural hypha in the middle layer of fungus gardens. Since enrichment timelines did not vary statistically for ^15^N and ^13^C, we visualized the overall temporal relationship by plotting standardized *Z*‐scores averaged across the nutrients (± SE). Significant Tukey test results within gongylidia tissue relative to day 0 are indicated with an asterisk (where **P < 0.01, ***P < 0.001), and significant differences (*P* < 0.05) between gongylidia and surrounding hypha tissue within days indicated with a cross (†). The dashed line connects gongylidia sampling days and the solid line connects hypha sampling days. We show an in vitro culture of leafcutter ant fungus cultivar for reference, even though the samples for analysis in this experiment were harvested in vivo directly from colonies.

**Figure 3:**
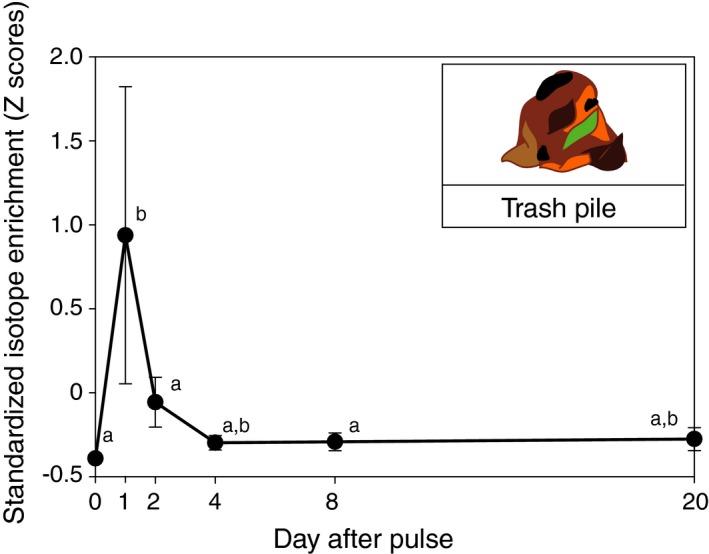
Waste‐disposal timelines of enriched substrates in the *A. colombica* farming symbiosis following harvest on day 1. Trash piles exhibited peak enrichment on the first day (means ± SE), indicating that large amounts of harvested substrate never reached the fungus garden. They also exhibited a slight enrichment uptick on day 20, suggesting gradual disposal of old enriched fungus. Standardized *Z*‐scores averaged across the nutrients are plotted because ^15^N and ^13^C timelines were not significantly different (Table 1). Letters indicate significantly different (Tukey test, *P* < 0.05) enrichment values across days.

*Fungus food:* We detected rapid integration of nutrients into edible gongylidia in the middle layer within two days of its harvest (Time × tissue interaction effects in Table 1, Fig. 2). Gongylidia were also significantly enriched relative to surrounding structural hypha by the second day (Table 1), and at equal levels for ^15^N and ^13^C (Table 1), indicating targeted conversion of both nutrients towards edible fungal food (Fig. 2). *Waste disposal:* Waste disposal also occurred rapidly on day 1 (Time effects in Table 1), with trash piles becoming maximally enriched at similar levels for ^15^N and ^13^C (Fig. 3). This indicates that foragers did not distinguish between nutrients when delivering substantial amounts of harvested resources directly to the trash. By day 20, we detected a slight uptick in trash enrichment (Fig. 3), potentially indicating the disposal of old fungus from the fungus garden.

### Nutrient allocation

**Figure 4:**
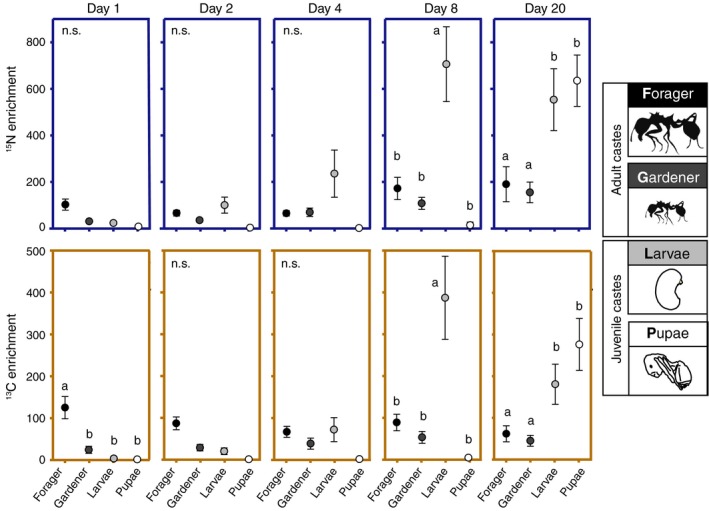
Comparing ^15^N and ^13^C enrichment across castes to evaluate the allocation hypothesis. Letters indicate significantly different (Tukey test, *P* < 0.05) enrichment values or groupings across castes within sampling days. The text ‘n.s.’ indicates no significant enrichment differences among castes within the sampling day. Head‐gaster tissue was analyzed for adult ants and whole bodies were analyzed for larvae and pupae. Enrichment means (± SE) are provided in units of μg ^15^N, ^13^C/g.

The allocation hypothesis was generally supported by caste‐specific enrichment dynamics (Nutrient × caste interaction effects in Table 1), with foragers assimilating significant levels of ^13^C, but not ^15^N when harvesting substrate (Fig. 4), and with larvae showing total enrichment levels that were higher for ^15^N than for ^13^C (Fig. 4). However, larvae on day eight (consuming enriched diet), and then pupae on day 20 (the aging cohort of enriched larvae) became significantly enriched for both ^15^N and ^13^C relative to adult castes (Fig. 4), indicating farming systems generally shunted nutrients towards ant colony growth (time × caste interaction effects in Table 1). Caste‐specific enrichment timelines also help refine nutrient transfer dynamics among nestmates. Specifically, an eight‐day lag from when foragers harvested enriched diet to when larvae and gardeners became enriched (Fig. 4) indicates these within‐garden castes did not directly assimilate resources regurgitated by returning foragers. Rather, they instead appeared to rely on cultivar‐derived resources.

### Nutrient processing

**Figure 5:**
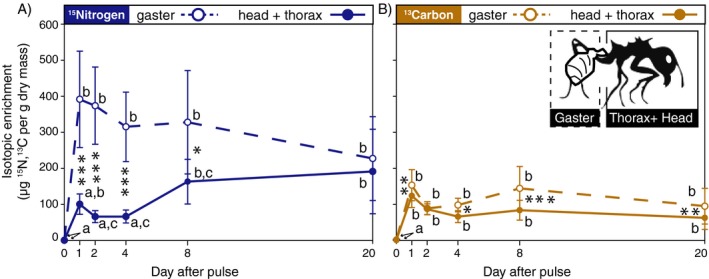
Testing forager‐first and fungus‐first models of nutrient processing in forager ants for (A) ^15^N‐enriched ammonium nitrate (blue lines) and (B) ^13^C‐enriched glucose (gold lines). Ants were subdivided prior to isotope analyses, to compare timelines of enrichment (means ± SE) reflecting nutrient transport in gaster tissue (dashed lines) and nutrient assimilation in head‐thorax tissue (solid lines). We used post‐hoc Tukey tests to interpret significant differences (*P* < 0.05) within tissues relative to day 0 (letters indicate significance groupings) and across tissues within sampling days (asterisks, where *P < 0.05, **P < 0.01, ***P < 0.001) indicate significant differences). Gaster tissue ^15^N‐enrichment on day 20 did not differ significantly from enrichment on day 0 (tukey result excluded for clarity).

We found mixed support for the hypothesis that foragers nutritionally bypass their gardens (*i.e.,* forager‐first processing). As evidence of assimilation during resource harvest, foragers had a significant head‐thorax pulse from day 0 to 1 for ^13^C, and a positive (although non‐significant) trend for ^15^N (Fig. 5). However, a more complex picture emerges considering this assimilation in the context of all nutrients ingested while foraging, as gaster enrichment timelines differed from those of head‐thorax tissue for both ^15^N and ^13^C (Time × tissue interaction effects for both nutrients in Table 1). Distinct ingestion‐assimilation timelines for ^13^C and ^15^N further indicate that the likelihood of forager‐first processing varies across nutrients. For instance, foragers appeared to bypass their cultivars to assimilate glucose as their head‐thorax tissues had consistently elevated ^13^C enrichment following consumption. The consistently higher ^13^C gaster enrichment (Fig. 5) is also consistent with known glucose processing and fat body storage dynamics. Processing dynamics for ammonium nitrate are more difficult to interpret, as foragers initially assimilated small fractions of ingested ^15^N and then gradually assimilated larger amounts over 20 d as it was simultaneously depleted from their gasters (Fig. 5).

Gardeners exhibited similar nutrient ingestion‐assimilation trends as foragers, for instance with consistently higher ^13^C enrichment in gaster tissue relative to head‐thorax tissue following ingestion (Appendix S3: Fig. S1), even as significant enrichment differences existed among these castes for both ^15^N and ^13^C (Caste effects for both nutrients in Table 1). These differences were likely driven by significantly delayed ingestion timeline of gardeners, as gardeners only exhibited significant assimilation for ^13^C eight days and ^15^N twenty days after the initial day 0 pulse (Appendix S3: Fig. S1).

## Discussion

This study clarifies the nutrient‐processing dynamics enabling leafcutter ants to convert harvested substrates into fungal food, and thus helps visualize how these farming systems unlock plant primary production as dominant herbivores across tropical ecosystems. Our results helped confirm unresolved nutritional hypotheses (e.g., nutrients are rapidly integrated into edible gongylidia), rule out others (e.g., foragers do not directly provision gardeners and larvae), and provide a template for disentangling others (e.g., the order of nutrient exchange between gongylidia, larvae, and gardeners). We further highlight how specific nutrients are transferred among symbiotic partners depending on their physiological requirements (e.g., allocating ^13^C in adult ants and ^15^N in developing brood) and metabolic processing capabilities (e.g., forager‐first assimilation of glucose, but not ammonium nitrate). We envision using this isotopic approach in field studies moving beyond identifying the substrates harvested by farming ants (Leal and Oliveira 2000, Seal and Tschinkel 2008) to mapping the underlying nutritional landscapes navigated by foragers.

### Ecology of farming productivity

Fungal cultivar genomes exhibit a variety of metabolic processing adaptations resulting from millions of years of co‐evolutionary selection as cultivated symbionts (De Fine Licht et al. 2014, Nygaard et al. 2016). We explore the in vivo performance consequences of this crop selection, quantifying conversion rates of harvested nutrients into fungal food. We found evidence that cultivars deliver rapid and targeted gongylidia production, with both nutrients shunted towards food production within 2 d, even as their overall downward processing rates differed within and across layers of the fungus garden. This rapid metabolic processing is consistent with our current understanding of the enzyme specialization of cultivars (De Fine Licht et al. 2010, Kooij et al. 2011, Seal et al. 2014), and with the enzyme vectoring by ants to detoxify (De Fine Licht et al. 2013) and digest (Moller et al. 2011) substrate even before it is deposited on gardens. Fast gongylidia production rates may also govern the high metabolic rates of gongylidia‐bearing fungi relative to less specialized cultivars of other attines that only produce hyphae (Shik et al. 2014b). Additionally, the capacity for fast substrate decomposition may have made ancestors of extant attine cultivars good symbiotic partners, despite their unremarkable nutritional qualities relative to other free‐living fungi (Mueller et al. 2001).

Despite the fundamental advantages of collective foraging, the task of provisioning ant nestmates with different nutritional requirements also provides complex challenges about which nutrients to harvest and in what blends (Dussutour and Simpson 2008a). Leafcutter foragers likely face even greater nutritional challenges as they provision completely unrelated fungal cultivars, saprophytes with very different nutritional requirements (Shik et al. 2016). And, while we hypothesized that ants would selectively dispose of less desirable crop producing nutrients prior to depositing substrate on the fungus garden, workers actually placed similar amounts of both nutrients directly in their trash piles (Fig. 3). Further study will be needed to determine whether this seemingly ‘wasted’ foraging effort stemmed from physical properties of agar diets, the high amount of available nutrients contained per gram of diet relative to a typical leaf fragment, or whether it was simply analogous to the large piles of unused leaf fragments often generated outside nest entrances by leafcutter colonies in nature (Wirth et al. 2003).

### Nutrient allocation

Despite the many ecological advantages of farming fungus (e.g., access to a stable resource supply), the nutritional challenges of a fungal diet can be inferred from the rarity of fungivory across the ant phylogeny (von Beeren et al. 2014). Our results provide evidence that leafcutter ants may overcome these challenges by targeted allocation of fungal‐derived nutrients to specific castes. Specifically, while brood were highly enriched for ^15^N, supporting a prediction of the allocation hypothesis, brood also had high ^13^C‐enrichment levels, supporting a general trend of allocating nutrients to colony growth. Further study will be needed to link specific nutrients fueling colony growth with the labeled compounds provided in diets, since larvae appeared to consume the metabolic byproducts of fungal cultivars rather than liquids supplied by returning foragers.

### Nutrient processing

Nutrient‐specific enrichment timelines of gasters (ingestion) and head‐thorax tissue (assimilation) shed light on the underlying metabolic processing dynamics. First, ^13^C‐enriched timelines support forager‐first assimilation of harvested glucose as their head‐thorax tissue remained consistently more enriched than pre‐harvest baseline over 20 d. Moreover, since their gasters remained even more ^13^C‐enriched than their head‐thorax tissues (Fig. 5), the ants likely converted much of the ingested glucose to glycogen and stored it in abdominal fat body cells (Roma et al. 2006). In contrast, the delayed assimilation of ^15^N until day 20 was potentially consistent with both fungus‐first and forager‐first hypotheses, although both explanations imply pre‐processing of ammonium nitrate by microbial symbionts. Specifically, ants may have relied on cultivars to convert ammonium nitrate into edible gongylidia, which they then consumed by day 20 (*fungus first*), or the ants' own digestive systems, aided perhaps by their recently characterized resident communities of symbiotic gut microbes (*i.e.,* Sapountzis et al. 2015), may have gradually metabolized the ammonium nitrate, making it available to their ant hosts over time (*forager first*). The forager‐first hypothesis seems likely, since the gradual transfer of ^15^N from the ants' alimentary canals to head‐thorax tissue (Fig. 5), implies a reliance on metabolic work performed by microbial gut symbionts. The fungus‐first hypothesis might thus be more aptly called the ‘symbiont‐first hypothesis’.

These ingestion‐assimilation results also help resolve a 40‐yr old debate in the attine literature about the primacy of fungus in leafcutter ant diets (Littledyke and Cherrett 1976, Stradling 1978, Wetterer 1994, Mueller et al. 2001, Silva et al. 2003, Rytter and Shik 2016). First, while Littledyke and Cherrett (1976) initially confirmed ingestion of plant sap by foraging leafcutter ants, they analyzed entire ant bodies and could thus not distinguish between assimilated nutrients, and liquids shared with nestmates or vectored directly to fungus gardens. Our results highlight the dynamic nature of resource‐exchange dynamics within leafcutter symbioses, as workers appear to nutritionally bypass their fungal cultivars depending on their ability to metabolize the ingested compound, and whether they are a caste that forages outside the nest. Thus, while our results highlight remarkable functional integration among symbiotic partners, they also highlight that fungal cultivars may only partially meet their farmers' nutritional needs. Moving forward, it will be important to explore how these production dynamics vary when these broad‐ranging generalist foragers encounter taxonomically (Wirth et al. 2003), nutritionally (Kooij et al. 2011), and biochemically (Howard 1988) diverse plant substrates, and when culitvars are farmed across ecological gradients (Mueller et al. 2011).
