# Supplementary material for: Disentangling nutritional pathways linking leafcutter ants and their co‐evolved fungal symbionts using stable isotopes
Source: Ecology. 2018 Aug 1;99(9):1999–2009. doi: 10.1002/ecy.2431 (PMC6174977; doi:10.1002/ecy.2431)
Supplement: Supplementary file 1 [file ECY-99-1999-s001.pdf]

Supporting Information. Disentangling nutritional pathways linking leafcutter ants and their co-evolved fungal symbionts using stable isotopes. Jonathan Z. Shik, Winnie Rytter, Xavier Arnan and Anders Michelsen. *Ecology.* 2018.

Appendix S1

Colony Establishment

In November 2015, we established queenless subcolonies from five queenright colonies of *Atta colombica* (Ac2012-32, Ac2012-31, Ac2009-42, Ac2012-1, Ac2011-3) in open plastic nest boxes (38 × 28 cm) with fluon-coated walls. Colonies were housed in a climate-controlled room (25°C, 70% RH, minimal daylight) at the Centre for Social Evolution, at the University of Copenhagen in Denmark. Minimal daylight (light through a window, but direct lights off unless somebody was working in the climate chamber) was used to mimic constant darkness in underground nests, and because direct sunlight in the foraging environment under a rainforest canopy is generally limited. For the next four months, subcolonies remained connected via tygon tubing to the queenright colonies, so workers could establish fungus garden while regulating their demography as an integrated satellite of the 'parent' colonies. Workers cultivated their fungus gardens under inverted plastic beakers fit with removable sampling windows (for ease of removing samples for isotope analysis) (Fig. 1A) and were provided with fresh bramble leaves, apple slices, and rice three times per week. In March 2016, a healthy subcolony (hereafter 'colony') was detached from each of the 5 parent colonies and designated for use in the feeding experiment. In this way, the parent colonies could be returned, uncontaminated with heavy isotopes, to the main leafcutter colony collection. Since much of the colony was embedded within the fungus garden, it was not possible to assess the demography of colonies until after the feeding experiment (Table S1).

Isotopically enriched diets

We modified the nutritionally defined protein:carbohydrate (1:3 and 3:1 P:C) ant diets of Dussutour and Simpson (2008) to be enriched for ^{13}C by adding D-glucose: $^{13}C_6H_{12}O_6$, (99 at% ^{13}C; Sigma-Aldrich) and ^{15}N by adding ammonium nitrate: $^{15}NH_4{}^{15}NO_3$, (98 at% ^{15}N; Sigma-Aldrich) (see Table S2 for itemized recipes). Diets were prepared by first boiling 1.6 g of agar (Sigma-Aldrich) in 100 ml of distilled water. Once this mixture cooled for a few minutes, it was combined with a mixture of egg powder (The Great American Spice Co.), whey protein (Bulk powders), calcium caseinate (Arla Foods), glucose (Sigma-Aldrich), Vanderzant vitamin mixture for insects (Sigma-Aldrich), and isotopically enriched compounds to yield 60 g/L protein and carbohydrates (Table S2). These ingredients were mixed in a blender, poured into petri dishes (15 g wet mass of diet per dish), and stored at 5°C.

As indicated by mass spectrometry, our enriched diets contained the following APE values: 1:3 P:C ($^{13}C_{APE}$ = 1.9 ± 0.1 SE & $^{15}N_{APE}$ = 6.9 ± 0.9 SE; n = 5 samples); 3:1 P:C ($^{13}C_{APE}$ = 2.4 ± 0.1 SE & $^{15}N_{APE}$ = 4.2 ± 0.4 SE; n = 6 samples). These isotope enrichment levels were

selected following a series of pilot experiments as the values that provided optimal resolution for tracing nutrient flow through the symbiosis. We also quantified '*initial diet harvest*' of enriched diet by colonies, measuring initial wet mass and final dry mass after 24h of ant foraging (remaining diet was dried at 60°C for 24h) and then estimating dry mass loss, from the dry/wet ratios of control diet. During the 24 hr foraging period, colonies selected P:C intake targets ranging from *ca.* 1:3 to 1:1 P:C (Table S2). Following Day 1 of the experiment, colonies were fed unenriched 1:3 and 3:1 P:C diets (Days 2-6) and bramble leaves (Days 7-20).

Isotopic sampling

We sampled leafcutter colonies on 6 days: 0, 1, 2, 4, 8, 20. Day 0 samples served as baseline 'natural abundance' measures and were collected just prior to providing colonies with enriched diets. At each sampling event, we collected (see Table S3 for planned sample sizes):

- gardener ants: small ants from within the fungus garden matrix
- medium foragers: collected outside the nest
- large foragers: collected outside the nest
- larvae: from the middle layer of the fungus garden
- pupae: from the middle layer of the fungus garden
- fungal hyphae: from top, middle, and bottom layers (with gongylidia removed)
- gongylidia: from the middle layer of the fungus garden
- trash pile

All components were dried at 60°C for ≥ 24 hours, homogenized, and then weighed into tin capsules. To prevent desiccation during gongylidia dissection, fungus samples were placed in plastic containers together with a wet cotton ball and stored in the climate-controlled room. To generate sufficient biomass for isotope analyses, gongylidia were pooled to yield one sample per colony per sampling day. Workers were anesthetized at 4°C and then divided into gaster samples and head-thorax samples. Each worker sample contained pooled gasters or head-thorax sections from 10 individuals (gardeners), or 3 individuals (medium, large foragers). We initially divided foragers between medium and large individuals for isotope analyses, but grouped them as a single 'forager' caste for statistical analyses (Wilson 1980). Since larvae and pupae did not have clearly defined body segments, they were sampled in their entirety.

For all figures and analyses, we calculated μg ^{15}N and μg ^{13}C enrichment values relative to the sample mass (see Methods section for details). In addition, we also calculated μg ^{15}N and μg ^{13}C enrichment values relative to the elemental mass of N and C per sample respectively. These means are provided for reference in Table S5A and S5B. We note that the shapes of the relationships remained the same (except for lower carbon enrichment in the trash pile, likely due to ^{12}C from old-uneriched fungus also being placed in the trash).

Some values measured for isotope enrichment before a given colony component encountered the labeled diet were slightly negative. These values were converted to zeroes prior

to statistical analyses. Isotopes could possibly be spread among workers through touching and grooming rather than nutritional food exchange via trophallaxis (da Silva et al. 2017). However, the staggered enrichment patterns in our study strongly suggest enrichment was due to substrate ingestion rather than simply an external contamination of ants occurring in an enriched fungus garden. For instance, pupae only became maximally enriched for both ^{13}C and ^{15}N on Day 20 (Fig. 4), and since pupae do not eat, the lag was due the gradual developmental transitions of larvae consuming enriched diet earlier in the experiment, rather than enrichment simply from being in the fungus garden.

Some Day 0 (natural abundance) samples of fungal hyphae from the middle and bottom layers of the fungus garden were slightly enriched for ^{15}N. This was likely due to a pilot experiment with enriched diets performed in the same climate-controlled room as the experimental colonies. Specifically, on February 18 (43 days before the actual experiment), we provided a subcolony (not used in the actual experiment) with agar-based diets enriched with ^{15}N-labeled ammonium nitrate in order to calibrate detection of enrichment dosages. Since the ammonium nitrate salt readily dissolved into solution when preparing the diets, some of the labeled ammonium cation likely evaporated in the humid room and landed on the colonies used in the experiment. As specified in the Methods section, we corrected for this by using Atom Percent Excess (APE) above Day 0 natural abundance when calculating per gram dry mass enrichment measures (*e.g.* $\mu g\ ^{15}N\ g\ dry\ mass^{-1}$).

BIBLIOGRAPHY

Dussutour, A. and S. J. Simpson. 2008. Description of a simple synthetic diet for studying nutritional responses in ants. Insectes Sociaux 55:329-333.

da Silva Camargo, R., C. Puccini, L. C. Forti, and C. A. Oliveira de Matos 2017. Allogrooming, self-grooming, and touching behavior: contamination routes of leaf-cutting ant workers using a fat-soluble tracer dye. Insects 8:59.

Wilson, E. O. 1980. Caste and division of labor in leaf-cutter ants (Hymenoptera: Formicidae: *Atta*) I. The overall pattern in *Atta sexdens*. Behavioral Ecology and Sociobiology 7:143-156.
