# Supplementary material for: Disentangling nutritional pathways linking leafcutter ants and their co‐evolved fungal symbionts using stable isotopes
Source: Ecology. 2018 Aug 1;99(9):1999–2009. doi: 10.1002/ecy.2431 (PMC6174977; doi:10.1002/ecy.2431)
Supplement: Supplementary file 2 [file ECY-99-1999-s002.pdf]

Supporting Information. Disentangling nutritional pathways linking leafcutter ants and their co-evolved fungal symbionts using stable isotopes. Jonathan Z. Shik, Winnie Rytter, Xavier Arnan, and Anders Michelsen. *Ecology*. 2018.

Appendix S2

Table S1: Final colony demography of *A. colombica* colonies measured on Day 20 following the feeding experiment. Colonies were frozen at -21°C prior to demographic sorting. The harvest (dry mass) of enriched substrate and fungal mass on Day 1 is also provided. All mass values dry mass in grams.

Colony ID	Enriched substrate harvest mass	Fungus mass	Worker		Larvae		Pupae	
			#	mass	#	mass	#	mass
Ac-2009-42	0.0505	15.78	10099	5.44	62	0.1203	1193	0.8189
Ac-2011-3	2.1003	16.04	16024	9.67	7	0.0231	723	0.4058
Ac-2012-1	0.2009	11.29	7947	3.06	128	0.0843	901	0.4055
Ac-2012-31	1.3744	18.7445	5435	2.8951	332	0.306	2265	1.5352
Ac-2012-32	0.9509	41.4587	18096	5.3745	124	0.2294	2912	2.4195

TABLE S2 We devised a modified version of the nutritionally defined protein:carbohydrate (P:C) diet of Dussutour and Simpson (2008) with a 60 g/L protein plus carbohydrate dilution that we enriched with the heavy isotopes ^{13}C and ^{15}N. For preparation details and heavy isotope enrichment levels, see methods and Appendix S1. Unenriched diets were similar, but replaced the ^{13}C-labeled glucose with unenriched glucose. Values in parentheses indicate the amount of protein provided by the ingredient as specified on ingredient labels. Small amounts of carbohydrates provided by egg powder (2.00%) and calcium caseinate (1.89%) were also incorporated into diet recipes. All amounts in grams (g), with 6 g protein + carbohydrates prepared in 100 ml of demineralized H_2O.

Ingredients	Enriched diets		Regular diets	
P:C ratio<br>Final H_2O volume (mL)	3:1<br>100	1:3<br>100	3:1<br>100	1:3<br>100
Dried egg powder	0.50 (0.24)	0.50 (0.24)	1.80 (0.85)	1.80 (0.85)
Whey protein	2.50 (2.02)	0.75 (0.60)	2.16 (1.74)	0.39 (0.31)
Calcium caseinate	2.50 (2.23)	0.75 (0.67)	2.16 (1.93)	0.39 (0.35)
Glucose	0 (0)	3.20 (0)	1.42 (0)	4.45 (0)
D-Glucose-1-^{13}C	1.43 (0)	1.24 (0)	0.00 (0)	0.00 (0)
Ammonium nitrate-^{15}N	0.34 (0)	0.11 (0)	0.00 (0)	0.00 (0)
Agar	1.60 (0)	1.60 (0)	1.60 (0)	1.60 (0)
Vanderzant vitamin mixture	0.16 (0)	0.16 (0)	0.16 (0)	0.16 (0)

Table S3 Planned isotope sampling scheme. Actual sample sizes (see Table S4A,B) differ slightly because some samples did not provide readable isotope values and others (*e.g.* trash on some days) were unavailable during collection events. See Appendix S1 for details about sampling protocols. Large ants collected outside the nest were initially sampled as 'large' and 'medium' foragers, but were pooled into a 'forager' caste prior for statistical analyses.

Sample type	Subsample	Replicates x colony x day	Colonies	Sampling days	n subsample	N sample type
Gardener ants	Gaster	2	5	6	60	120
	Head-thorax	2	5	6	60	
Forager ants	Gaster	4	5	6	60	240
	Head-thorax	4	5	6	60	
Brood	Larvae	3	5	6	90	180
	Pupae	3	5	6	90	
Fungal hyphae	Top layer	3	5	6	90	270
	Middle layer	3	5	6	90	
	Bottom layer	3	5	6	90	
Fungal gongylidia	Middle layer	1	5	6	30	30
Trash		2	5	6	60	60
Total						900

TABLE S4A Sample mass-specific enrichment: Average isotope enrichment values in units of average µg ^{15}N per g dry weight (± SE) of sampled tissue per day (first averaged at the colony level) in *Atta colombica* colonies. Slightly negative values (indicating no detectable enrichment) were converted to zeroes prior to calculation of averages, analyses, and construction of figures.

^{15}N Raw Means (µg ^{15}N g DW^{-1} ± SE)												
**Sample**	**Type**	**Part**	**Day 1**		**Day 2**		**Day 4**		**Day 8**		**Day 20**	
			**N**	**Mean ± SE**	**N**	**Mean ± SE**	**N**	**Mean ± SE**	**N**	**Mean ± SE**	**N**	**Mean ± SE**
Fungus	Structure	Top	15	40.9 ± 17.0	15	100.3 ± 36.6	10	258.4 ±77.7	15	248.6 ± 119.8	15	59.6 ±36.3
		Middle	15	73.6 ± 35.816	15	141.3 ± 91.6	10	118.0 ± 97.4	15	125.3 ± 80.1	15	162.9 ± 99.5
		Bottom	15	17.3 ± 11.5	15	5.8 ± 5.7	10	31.0 ± 22.3	14	11.2 ± 11.2	15	24.5 ± 23.9
	Gongylidia	Middle	5	280.7 ± 102.1	5	628.9 ± 195.6	5	441.8 ± 177.0	5	407.9 ± 200.2	5	217.2 ± 112.2
Ant	Forager	Gaster	17	391.5 ± 133.7	20	373.7 ± 107.1	18	315.0 ± 96.2	17	327.9 ± 143.4	19	227.0 ± 116.1
		Body	17	100.4 ± 28.4	20	66.1 ± 16.6	18	66.2 ± 17.8	17	163.0 ± 61.9	19	191.2 ± 117.0
	Gardener	Gaster	10	60.6 ± 26.7	10	102.9 ± 51.2	10	195.0 ± 73.8	10	249.3 ± 105.3	10	168.6 ± 70.8
		Body	10	30.8 ± 9.2	10	35.8 ± 12.4	10	69.6 ± 26.3	10	108.0 ± 38.4	10	155.0 ± 65.0
Brood	Larvae		15	23.5 ± 5.6	14	110.9 ± 42.8	15	235.4 ± 136.5	12	717.5 ± 246.3	15	553.6 ± 247.8
	Pupae		13	8.0 ± 1.8	15	3.6 ± 2.2	15	1.3 ± 0.5	15	14.3 ± 13.0	15	634.9 ± 190.0
Trash			10	457.1 ± 279.8	10	137.4 ± 73.1	5	28.1 ± 18.2	7	39.5 ± 24.2	5	49.0 ± 33.0

TABLE S4B Sample mass-specific enrichment: Average isotope enrichment values in units of average µg ^{13}C per g dry weight (± SE) of sampled tissue per day (first averaged at the colony level) in *Atta colombica* colonies. Slightly negative values (indicating no detectable enrichment) were converted to zeroes prior to calculation of averages, analyses, and construction of figures.

^{13}C Raw Means (µg ^{13}C g DW^{-1} ± SE)												
**Sample**	**Type**	**Part**	**Day 1**		**Day 2**		**Day 4**		**Day 8**		**Day 20**	
			**N**	**Mean ± SE**	**N**	**Mean ± SE**	**N**	**Mean ± SE**	**N**	**Mean ± SE**	**N**	**Mean ± SE**
Fungus	Structure	Top	15	20.4 ± 9.3	15	35.3 ± 12.5	10	37.2 ± 13.2	15	42.4 ±20.8	15	9.1 ± 3.4
		Middle	15	109.6 ± 42.9	15	162.2 ± 70.6	10	138.5 ± 72.3	15	108.4 ± 29.4	15	22.7 ±16.2
		Bottom	15	24.7 ± 17.6	15	22.6 ± 13.4	10	40.9 ± 24.1	14	18.4 ± 7.6	15	34.8 ± 17.8
	Gongylidia	Middle	5	51.1 ± 15.0	5	335.8 ± 145.9	5	325.1 ± 205.3	5	221.5 ± 62.9	4	61.1 ± 29.2
Ant	Forager	Gaster	17	153.1 ± 43.3	20	89.1 ± 18.1	18	97.9 ± 18.9	17	144.4 ± 60.6	19	94.9 ± 49.4
		Body	17	123.6 ± 32.1	20	86.7 ± 14.9	18	66.0 ± 16.7	17	83.8 ± 27.6	19	62.5 ± 31.8
	Gardener	Gaster	10	32.3 ± 16.2	10	46.2 ± 18.3	10	62.9 ± 31.3	10	97.8 ± 37.3	10	83.9 ± 36.5
		Body	10	24.3 ± 11.1	10	28.7 ± 11.1	10	38.9 ± 19.5	10	53.3 ± 20.8	10	44.9 ± 18.9
Brood	Larvae		15	3.5 ± 0.7	14	22.9 ± 10.6	15	72.3 ± 30.3	12	408.1 ± 163.1	15	180.6 ± 89.5
	Pupae		13	1.4 ± 0.7	15	0.8 ± 0.6	15	1.9 ± 0.9	15	4.6 ± 1.8	15	276.1 ± 108.2
Trash			10	607.0 ± 440.0	10	123.8 ± 39.3	5	52.6 ± 14.7	7	41.8 ± 15.6	5	43.1 ± 17.2

TABLE S5A Sample element mass-specific enrichment: Average isotope enrichment values in units of average µg ^{15}N per g N weight (± SE) in sampled tissue per day (first averaged at the colony level) in *Atta colombica* colonies. Slightly negative values (indicating no detectable enrichment) were converted to zeroes prior to calculation of averages, analyses, and construction of figures.

^{15}N Raw Means (µg ^{15}N g N^{-1} ± SE)														
**Sample**	**Type**	**Part**	**Day 1**		**Day 2**		**Day 4**		**Day 8**		**Day 20**	
			**N**	**Mean ± SE**	**N**	**Mean ± SE**	**N**	**Mean ± SE**	**N**	**Mean ± SE**	**N**	**Mean ± SE**
Fungus	Structure	Top	15	1,210.1 ± 474.2	15	2,789.3 ± 960.6	10	6,229.7 ± 2,074.3	15	6,835.3 ± 2,944.1	15	1,578.7 ± 973.8
		Middle	15	3,577.2 ± 1,877.5	15	5,699.6 ± 3,480.6	10	4,794.5 ± 3,612.8	15	4,695.3 ± 2,552.3	15	4,140.8 ± 2,430.2
		Bottom	15	802.6 ± 505.7	15	258.4 ± 252.1	10	1,931.7 ± 1,470.4	14	419.1 ± 419.1	15	737.8 ± 696.5
	Gongylidia	Middle	5	8,467.1 ± 2,993.1	5	18,220.8 ± 5,591.2	5	12,499.2 ± 4,882.9	5	11,851.1 ± 5,643.2	5	4,970.2 ± 2,498.9
Ant	Forager	Gaster	17	4,680.4 ± 1,559.8	20	4,466.7 ± 1,246.7	18	3,519.2 ± 973.2	17	4,251.6 ± 1,611.8	19	3,056.6 ± 1,651.1
		Body	17	835.4 ± 229.8	20	571.8 ± 138.2	18	575.3 ± 150.3	17	1,495.5 ± 565.1	19	1,735.1 ± 1,081.6
	Gardener	Gaster	10	954.6 ± 409.3	10	1,532.6 ± 716.1	10	3,005.6 ± 1,150.1	10	4,124.4 ± 1,647.1	10	2,665.3 ± 1,063.0
		Body	10	285.6 ± 83.7	10	331.0 ± 113.2	10	665.1 ± 248.8	10	1,019.4 ± 358.8	10	1,461.2 ± 605.2
Brood	Larvae		15	428.5 ± 105.8	14	1,964.0 ± 729.0	15	3,877.5 ± 2,269.3	12	11,921.0 ± 3,988.2	15	9,005.2 ± 4,065.6
	Pupae		13	124.7 ± 30.7	15	52.8 ± 30.7	15	20.0 ± 7.4	15	201.9 ± 185.4	15	9,079.0 ± 2,799.8
Trash			10	13,462.2 ± 7,350.5	10	5,337.8 ± 2,885.8	5	921.8 ± 599.3	7	1,472.4 ± 927.0	5	1,850.6 ± 1,163.7

TABLE S5B Sample element mass-specific enrichment: Average isotope enrichment values in units of average µg ^{13}C per g C weight (± SE) in sampled tissue per day (first averaged at the colony level) in *Atta colombica* colonies. Slightly negative values (indicating no detectable enrichment) were converted to zeroes prior to calculation of averages, analyses, and construction of figures.

^{13}C Raw Means (µg ^{13}C g C^{-1} ± SE)												
**Sample**	**Type**	**Part**	**Day 1**		**Day 2**		**Day 4**		**Day 8**		**Day 20**	
			**N**	**Mean ± SE**	**N**	**Mean ± SE**	**N**	**Mean ± SE**	**N**	**Mean ± SE**	**N**	**Mean ± SE**
Fungus	Structure	Top	15	43.6 ± 20.0	15	76.3 ± 27.5	10	83.7± 30.4	15	87.5 ± 43.5	15	18.8 ± 7.0
		Middle	15	237.4 ± 93.2	15	363.3 ± 160.6	10	314.0 ± 166.1	15	234.8 ± 62.3	15	47.1 ± 33.1
		Bottom	15	52.8 ± 37.2	15	50.7 ± 30.4	10	92.0 ± 54.4	14	38.7 ± 16.7	15	75.3 ± 37.9
	Gongylidia	Middle	5	113.9 ± 33.3	5	755.1 ± 321.0	5	751.6 ± 471.9	5	508.0 ± 149.6	4	137.7 ± 62.6
Ant	Forager	Gaster	17	283.2 ± 83.4	20	166.7 ± 35.5	18	187.9 ± 37.6	17	257.0 ± 106.1	19	172.0 ± 88.0
		Body	17	219.9 ± 53.3	20	164.5 ± 29.8	18	127.1 ± 32.7	17	154.6 ± 49.5	19	117.8 ± 59.5
	Gardener	Gaster	10	56.4 ± 27.9	10	82.2 ± 34.7	10	115.2 ± 58.1	10	166.4 ± 62.1	10	144.9 ± 62.7
		Body	10	45.0 ± 20.4	10	53.4 ± 21.0	10	74.9 ± 38.0	10	95.9 ± 36.0	10	84.0 ± 34.8
Brood	Larvae		15	7.4 ± 1.5	14	48.1 ± 22.5	15	148.6 ± 62.2	12	806.3 ± 305.6	15	374.6 ± 185.3
	Pupae		13	3.0 ± 1.5	15	1.7 ± 1.3	15	3.7 ± 1.7	15	9.3 ± 3.7	15	576.8 ± 227.5
Trash			10	1,266.7 ± 916.9	10	281.6 ± 89.5	10	110.5 ± 30.5	5	87.4 ± 32.4	7	95.4 ± 39.4
