# Supplementary material for: Disentangling nutritional pathways linking leafcutter ants and their co‐evolved fungal symbionts using stable isotopes
Source: Ecology. 2018 Aug 1;99(9):1999–2009. doi: 10.1002/ecy.2431 (PMC6174977; doi:10.1002/ecy.2431)
Supplement: Supplementary file 3 [file ECY-99-1999-s003.pdf]

Supporting Information. Disentangling nutritional pathways linking leafcutter ants and their co-evolved fungal symbionts using stable isotopes. Jonathan Z. Shik, Winnie Rytter, Xavier Arnan, and Anders Michelsen. *Ecology*. 2018.

Appendix S3

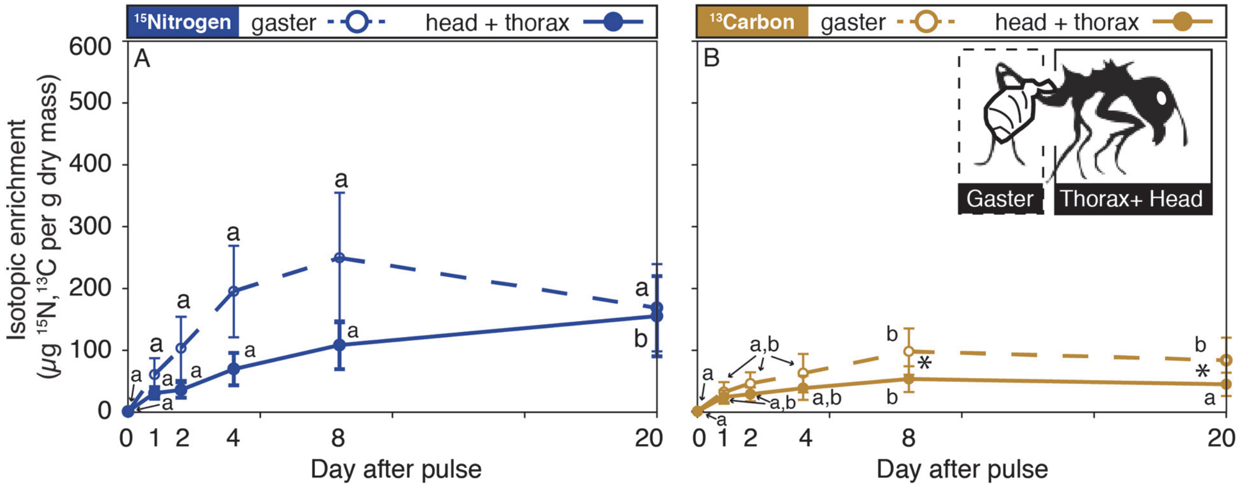

FIGURE S1 Gardener ants exhibited similar nutrient processing trends as foragers, except with delayed ingestion. Ants gasters (dashed lines) were analyzed separately from head-thorax tissue (solid lines) for enrichment of A) ^{15}N-enriched ammonium nitrate (blue lines) and B) ^{13}C-enriched glucose (gold lines). Tukey test comparisons, indicated by letter groupings, show significant differences relative to day 0 ($p < 0.05$) across days within tissues (gaster or head-thorax), and comparisons indicated by asterisks (where *$p < 0.05$, **$p < 0.01$, ***$p < 0.001$), indicate significant differences within days, but across tissues.
